# Converging functional phenotyping with systems mapping to illuminate the genotype–phenotype associations

**DOI:** 10.1093/hr/uhae256

**Published:** 2024-09-09

**Authors:** Ting Sun, Zheng Shi, Rujia Jiang, Menachem Moshelion, Pei Xu

**Affiliations:** Key Laboratory of Specialty Agri-product Quality and Hazard Controlling Technology of Zhejiang Province, College of Life Sciences, China Jiliang University, Hangzhou 310018, P.R. China; Key Laboratory of Specialty Agri-product Quality and Hazard Controlling Technology of Zhejiang Province, College of Life Sciences, China Jiliang University, Hangzhou 310018, P.R. China; Key Laboratory of Specialty Agri-product Quality and Hazard Controlling Technology of Zhejiang Province, College of Life Sciences, China Jiliang University, Hangzhou 310018, P.R. China; The Robert H. Smith Institute of Plant Sciences and Genetics in Agriculture, The Robert H. Smith Faculty of Agriculture, Food and Environment, The Hebrew University of Jerusalem, Rehovot 76100, Israel; Key Laboratory of Specialty Agri-product Quality and Hazard Controlling Technology of Zhejiang Province, College of Life Sciences, China Jiliang University, Hangzhou 310018, P.R. China

## Abstract

Illuminating the phenotype–genotype black box under complex traits is an ambitious goal for researchers. The generation of temporally or spatially phenotypic data today has far outpaced its interpretation, due to their highly dynamic nature depending on the environment and developmental stages. Here, we propose an integrated enviro-pheno-geno functional approach to pinpoint the major challenges of decomposing physiological traits. The strategy first features high-throughput functional physiological phenotyping (FPP) to efficiently acquire phenotypic and environmental data. It then features functional mapping (FM) and the extended systems mapping (SM) to tackle trait dynamics. FM, by modeling traits as continuous functions, can increase the power and efficiency in dissecting the spatiotemporal effects of QTLs. SM could enable reconstruction of a genotype–phenotype map from developmental pathways. We present a recent case study that combines FPP and SM to dissect complex physiological traits. This integrated approach will be an important engine to drive the translation of phenomic big data into genetic gain.

The phenomics era is witnessing high-throughput, high-dimensional phenotyping of a massive set of traits from various environments [1]. Specifically, functional physiological phenotyping (FPP, Table 1) referring to physiology-based, high-throughput and nondestructive phenotyping enables simultaneous and continuous measurement of plants alongside their surrounding soil and atmospheric conditions [2]. Since the key traits that FPP targets, e.g., photosynthesis and transpiration rates (TRs), are yield-associated, FPP has become an important tool in both basic research and plant (pre)breeding [3]. However, FPP's utility in most current practices rarely extends to the point of unraveling the underlying genetic networks and molecular pathways of the traits. One major reason for this limitation is that current genetic pipeline is not powerful enough to handle the booming, multi-factorial, time-series data generated by FPP [4, 5]. Consequently, researchers face the daunting challenge of maximizing data interpretation capacity and bridging the phenotype–genotype gap. In this perspective paper, we argue that, to tackle this challenge effectively, the advances of FPP should be merged with the latest advancements in genetics for complex systems such as functional mapping (FM, Table 1) and systems mapping (SM, Table 1).

**Table 1:** Explanations of key terms (in order of appearance)

Term	Explanation
Functional physiological phenotyping (FPP)	FPP is a non-invasive, high-throughput phenotyping method grounded in plant physiology, offering continuous measurements of both the plant and its surroundings. In contrast to traditional structural phenotyping, FPP focuses on monitoring the plant's intrinsic physiological processes, offering a more fundamental and sensitive method for assessing plant responses to environmental changes
Functional mapping (FM)	FM identifies dynamic quantitative trait loci (QTLs) and analyzes their impact on trait development and expression by estimating the curve parameters for each QTL genotype and comparing these parameters across different genotypes. Compared to conventional static mapping, FPP offers a quantitative and testable framework for assessing the interplay between gene actions or interactions and developmental changes
Systems mapping (SM)	SM identifies QTLs that control developmental interactions of traits, the temporal pattern of QTLs expression during development, as well as the genetic determinants that control developmental switches. Unlike FM, which concentrates on specific processes within a system, SM aims to comprehend and manage complex systems by visualizing interactions and dependencies. It examines the various components that make up the system through developmental regulation and links them using biologically meaningful differential equations
Linkage mapping	Linkage mapping is a genetic technique used to determine the locations of genes or genetic markers associated with specific traits by analyzing recombination frequencies between them. It relies on the principle that genes situated close to each other on the same chromosome are likely to be inherited together. This method is particularly effective for identifying linked genetic markers in structured populations. Linkage mapping may be limited by the number of recombination events, often leading to large confidence intervals for QTL locations
Association mapping	Association mapping is a strategy that leverages past linkage disequilibrium to establish a direct connection between QTLs and phenotypes, thereby unveiling genetic associations. It offers high resolution by using historical recombination events in natural populations to identify specific genetic variants. However, it demands very large sample sizes to detect associations with small effect sizes and requires advanced statistical methods and substantial computational resources for data analysis
QTL mapping	QTL mapping is a comprehensive genomic analysis used to deduce the relationship between genotype at various locations in the genome and the phenotypes associated with a set of quantitative traits. This process explores the number, genomic positions, effects, and interactions of QTL. The primary aim of QTL mapping is to pinpoint the chromosomal regions that exert a significant influence on the variation observed in quantitative traits within a population. Traditional statistical methods for QTL mapping, which analyze phenotypic data at a single time point, are often insufficient for revealing the genetic control of developmental processes. These approaches fail to account for the developmental aspects of trait formation
Expectation–Maximization (EM) algorithm	In statistics, the EM algorithm is an iterative technique used to calculate maximum likelihood or maximum a posteriori estimates of parameters in statistical models that include latent variables. This process alternates between an expectation (E) step, where the expected log-likelihood is computed, and a maximization (M) step, where parameters are updated to maximize this expected log-likelihood. These newly estimated parameters are then used to determine the distribution of latent variables in the subsequent E step
Maximizing likelihood	Maximizing likelihood is a fundamental principle in the fields of statistics and machine learning. It involves identifying the parameter values within a statistical model that result in the greatest likelihood function value. The likelihood function assesses the probability of observing the given data given the assumed statistical model and its parameters
Varying-coefficient regression	Varying-coefficient regression, also referred to as locally weighted scatterplot smoothing, is a statistical approach that adjusts regression coefficients according to specific covariate values in situations where variable relationships are not assumed to be uniform across the dataset
Markov Chain Monte Carlo	In statistics, Markov chain Monte Carlo (MCMC) methods are algorithms for sampling from a probability distribution. By constructing a Markov chain with the desired distribution as its equilibrium state and recording its states, one can obtain a sample that approximates the target distribution better with more steps
Hierarchical modeling	Hierarchical modeling, also known as multilevel modeling or hierarchical linear modeling (HLM), is a statistical method for analyzing data with a nested structure. It offers flexibility to model complex relationships and dependencies in the data by addressing multiple levels of variability
Legendre polynomials	Legendre polynomials are classical orthogonal polynomials that satisfy a second-order linear differential equation. This equation naturally emerges when solving initial boundary value problems in three-dimensional situations with spherical symmetry. Legendre Polynomials, or Legendre functions of the first kind, provide solutions to this differential equation
Structured antedependence model	In time series analysis, a structured antedependence model is a statistical technique applied to characterize and model the autocorrelation patterns within a time series, especially when these patterns demonstrate specific antedependence structures. Antedependence, in this context, signifies a pattern where observations are positively correlated at shorter intervals and negatively correlated at longer intervals
Introgression line (IL)	An IL population comprises individuals resulting from a controlled breeding between two distinct species or varieties, often within the same genus. Its main goal is to study the transmission of specific genes or traits from one parent to another. This involves a repetitive process of backcrossing the hybrid offspring to one of the parental lines while focusing on the trait of interest
Conditional QTLs	Conditional QTLs are specific genomic regions that affect a particular trait or phenotype only under certain conditions or in the presence of other genetic or environmental factors. Their impact on the trait is context-dependent, varying based on the levels of other interacting genes, environmental conditions, or specific genetic backgrounds
Ordinary differential equations (ODEs)	ODEs, which stands for ordinary differential equations, are a class of differential equations that are dependent on a single independent variable. The use of the term "ordinary" distinguishes them from partial differential equations, which can involve more than one independent variable

**Figure 1:**
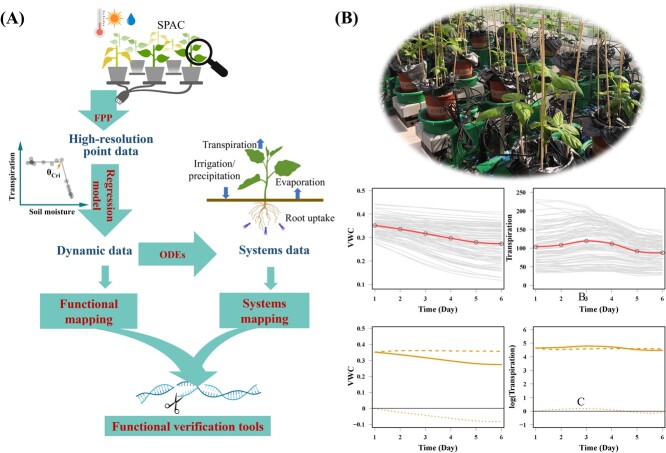
Principle of the integrated enviro-pheno-geno functional approach and an example showing combined FPP–SM approach to analyze the interaction of TR and VWC. (A) Integrated Enviro-pheno-geno functional approach is for systematically and dynamically understanding plant physiology, their interaction with the environment, and the underlying dynamic genes/QTLs as well as their networks. FPP represents functional physiological phenotyping. As depicted in the graph, lysimeter-based FPP enables simultaneous and continuous measurement of plant and environmental parameters within the soil–plant–atmosphere continuum (SPAC). We present an instance of dynamic data points for TR taken at 3-min intervals. Fitting dynamic data with mathematical models like regression models can convert the scattered data points into a cohesive dataset. For instance, using piecewise functions to fit the midday transpiration-soil VWC response curve, one can obtain the critical soil water content (θ_cri_) for plant stomatal regulation to adapt to drought. Functional mapping (FM) approach integrates the mathematical aspect of trait development to estimate the net genetic effect of each locus that changes over time, increasing the power and efficiency of QTL analysis. Time-series measurements of a trait can be fitted to a biologically meaningful curve and integrated into FM. When there are mutual interactions between multiple dynamic traits, it is necessary to take them into account within a framework that encompasses the entire system. The ODEs are used for depicting how one component interacts dynamically with others in a complex system that includes multiple plant physiological processes, such as plant transpiration, soil evaporation, root water uptake, and irrigation/precipitation in the SPAC. Systems mapping (SM) extends the dynamic idea of functional mapping to dissect the phenotype into its interrelating components based on design principles and then map QTLs that determine each of the components and their interactions. (B) FPP datasets of 32 cowpea genotypes were used for genotype–phenotype analysis. Three plants of each genotype were grown on the PlantArray phenotyping platform in a pot filled with Profile Porous Ceramic substrate, with plastic film covering the soil surface to prevent evaporation. Five biological replicates were included for each genotype. The system recorded the TR and VWC of each pot every 3 minutes for six consecutive days as the plants experienced progressive soil drought due to water withholding, which were shown as grey lines. By denoting *X* and *Y* as the TR and VWC at the time *t*, respectively, a system of ODEs is constructed. The mean vectors of VWC and TR were shown as red lines. The solid lines in the graph illustrate the average fits of VWC and TR with their interactive effect, while the dashed lines show the average fit of these variables without the interactive effect. The dotted lines indicate the net interactive effects.

## FPP–FM as an effective tool for addressing trait dynamics in forward genetics

In efforts of attributing phenotypes to genotypes, which is essential for translating phenotyping advancements into genetic gain, the dynamic of physiological traits and their sensitivity to subtle environmental fluctuations present significant challenges [3, 6]. Despite significant progress in genetically unraveling diverse physiological traits, including canopy temperature and water use efficiency, as well as high-throughput image-based traits over the past decade [7–9], forward genetic analysis remains a rate-limiting step, with few methodological innovations. The traditional regression model-based genetic mapping approaches, such as linkage mapping for pedigreed populations and association mapping for natural populations, continue to dominate QTL mapping, with the disadvantage of disregarding the dependency among time-series data and treating them independently, resulting in a time-consuming process that fails to capture the dynamic structure and pattern of the trait formation [10].

To address this issue, the FM approach has been coupled with FPP (Fig. 1A). FM is a statistical framework to map genes controlling dynamic biological process of complex traits [11, 12]. In this framework, time-series trait measurements are fitted to a biologically meaningful curve, and a mixture model is fitted using expectation–maximization algorithm through maximizing likelihood (Table 1), followed by hypothesis testing the association significance. Several modeling strategies, such as Varying-Coefficient Regression, Markov Chain Monte Carlo, and hierarchical modeling (Table 1), have been implemented into the FM framework. Due to the integration of the mathematical aspect of trait formation to estimate the genetic effect of each locus that changes over time, it increases the power and efficiency of QTL analysis. For example, Jiang *et al*. [13] unraveled the genetic architecture of N-induced phenotypic plasticity in wheat by integrating FM and high-throughput spectrometer-based phenotyping. Yield-related canopy architecture in a natural population of wheat was continuously measured over the growing cycle. In contrast to traditional association analysis using a linear mixed effect model, a joint logistic and exponential equation was used to model the mean vector by genotype-dependent parameters for different traits under different treatments. This integration enabled the decomposition of the temporal pattern of genetic effects on N- and microenvironment-induced phenotypic plasticity, highlighting the phenological landscape of genetic effects exerted by individual QTLs, as well as their interactions with N-induced signals and with canopy measurement angles. More recently, Pandey *et al*. [14] developed a joint FPP–FM framework within the maximum likelihood context and implemented with Legendre polynomial and structured antedependence model (Table 1), to map multi-phased physiological data. The functional trait, TR, was continuously and simultaneously measured for each line of a tomato introgression line population, using a lysimeter-based FPP system at a 3-minute frequency. Since the FPP–FM approach overcomes the overfitting or underfitting of time-series data, it detected seven QTLs, including a major conditional QTL that was specifically expressed during the rehydration phase. In comparison, traditional mapping methods detected only two QTLs from the same data set. Furthermore, Pandey *et al*. [14] conducted a simulation analysis comparing the powers of FPP–FM and traditional mapping methods (ANOVA) for IL populations. ANOVA effectively detected significant QTLs in uncorrelated data with large sample sizes and high heritability. However, its power for detecting QTLs in time-correlated data was notably lower (0.34–0.61). In contrast, FPP–FM demonstrated greater efficacy in identifying QTLs in time-correlated data, while its performance in handling time-uncorrelated data was comparable to ANOVA.

## Coupled FPP and SM for effectively addressing trait complexity

In a dynamic environment, various physiological processes not only exhibit significant levels of dynamism but also interact with each other, forming a complex system [15]. For instance, under constant vapor pressure deficit conditions, the net photosynthesis rate (A_n_) and TR or stomatal conductance (g_s_) typically respond synchronously to changes in leaf temperature from low to high. However, at elevated temperatures, there may be a decoupling between A_n_ and TR or g_s_. Stomata and mesophyll membranes can strategically collaborate to enhance transpiration cooling and CO_2_ supply, thereby mitigating heat stress on leaf photosynthetic function, although this synergy may result in reduced water-use efficiency [16]. Compared to structural traits, functional traits show more intense and rapid responses to the environment and interactions between each other, posing new challenges in data analysis. The FPP–FM approach represents a leap from static trait mapping to dynamic trait mapping, but it still falls short when mapping complex multifactorial traits that depend on interactions with other loci. FM has recently been upgraded to SM, which treats complex traits as dynamic systems composed of interactive components linked through ordinary differential equations (ODEs), which have proved useful in depicting how one component interacts dynamically with others (Fig. 1B). By dissolving a complex trait into its underlying components based on structural, biochemical, physiological, and developmental principles, the implementation of ODEs into SM can delineate QTLs governing the interconnections of different components [17–19]. Whereas not initially developed in plant systems, applications of SM have successfully analyzed complex plant traits such as biomass production and identified QTLs governing the interconnections between different components. In one study with soybean, QTLs displaying different temporal patterns for three organs were identified, and the functional relationships among leaf, stem, and root biomass were determined [18]. This study integrated the coordination and optimization model to study the pattern of biomass partitioning by incorporating the allometric scaling theory into a system of ODEs, where biomasses of different organs as well as the constant and exponent power of an organ biomass scaling as whole-plant biomass and the rate of eliminating ageing leaves or root hairs were accounted for. Gong *et al*. [20] constructed and implemented a nonlinear mixed mapping framework into SM to decompose the genetic mechanisms underpinning multiphasic growth changes between stem diameter and stem height in tress. QTLs harboring candidate genes potentially regulating ecological interactions between stem apical and lateral growth were identified. Wen *et al*. [21] recently extended SM to map developmental modularity, a system that comprises multiple developmental traits interacting either coordinately or competitively. These reports demonstrate the unique suitability of incorporating SM for drawing a global picture of the formation and development of higher-order complex traits.

To date, SM has not been formulated to high-throughput physiological traits in plants, but the idea of coupling the two holds enormous promise to simultaneously decompose many developmentally and environmentally-related components that jointly form the endpoint complex traits. By considering *n* physiological traits, we develop a general theoretical framework using an *n*-dimensional system of ODEs to model the dynamic change of physiological trait, expressed as


$$ \frac{dp_i}{dt}={f}_i\left({p}_i:{\theta}_i\right)+\sum_{i^{\prime }=1,{i}^{\prime }=i}^n{f}_{i\mid{i}^{\prime }}\left({p}_{i^{\prime }}:{\theta}_{i{i}^{\prime }}\right),i=1,\dots, n $$

where the change rate for each physiological trait *i* (*i* = 1, . . ., *n*) per unit time is split into two parts: the independent function ${f}_i\left({p}_i:{\theta}_i\right)$ represents the independent change in the trait that that occurs when the target trait is in isolation and the dependent function ${f}_{i\mid{i}^{\prime }}\left({p}_{i^{\prime }}:{\theta}_{i{i}^{\prime }}\right)$ represents how the independent change in the trait is influenced by other traits. The two functions can be fitted by nonparametric approaches, such as B-spline or Legendre orthogonal polynomials. The type and strength of interaction between traits is measured by the dependent parameters. When genetic background such as population structure and kinship need to be considered, the framework only needs to be implemented with fixed and random effect parameters.

As a proof of concept, here we present the analysis of the inter-dependence of TR, a plant physiological trait, and volumetric soil water content (VWC), an environmental parameter tightly associated with TR, in the plant–soil–atmosphere system. We utilized data from 32 cowpea genotypes grown on a lysimetric FPP system (Fig. 1B). In the experiment, we grew three plants of each genotype in a pot, with plastic film covering the soil surface to prevent evaporation. The system recorded the TR and VWC of each pot every 3 minutes for six consecutive days as the plants experienced progressive soil drought due to water withholding. By denoting *X* and *Y* as the TR and VWC at the time *t*, respectively, which allows us to construct a system of ODEs, expressed as


$$ \left\{\begin{array}{@{}c}\frac{dX}{dt}={f}_x\left(X:{\theta}_x\right)+{f}_{x\mid y}\left(Y:{\theta}_{x\mid y}\right)\\[2pt] {}\frac{dY}{dt}={f}_y\left(Y:{\theta}_y\right)+{f}_{y\mid x}\left(X:{\theta}_{y\mid x}\right)\end{array}\right. $$

where the change rate of the TR over time is decomposed into the independent function, *f*_x_, specified by unknown parameters ${\theta}_x$, and the dependent function *f*_x|y_, specified by unknown parameters ${\theta}_{x\mid y}$. The change rate of the VWC over time is decomposed into the independent function, *f*_y_, specified by unknown parameters ${\theta}_y$, and the dependent function *f*_y|x_, specified by unknown parameters ${\theta}_{y\mid x}$. As a result, the fitted VWC decreased gradually over time, due to water loss from leaf transpiration, while the fitted TR showed a slight increase during the initial days of water withholding when VWC was still relatively abundant, before declining from the fourth day after water withholding (Fig. 1B). By incorporating genome-wide DNA markers, the SM identified 24 significant SNPs (FDR < 0.05) across the genome to affect the relative contributions of competition and cooperation between VWC and TR (Table S1).

The two-phased TR pattern depending on drought strength during gradual soil water depletion has been observed across various species. For instance, Xu and Zhou [22] found that moderate water deficits positively affected stomatal number, which correlated with increased stomatal conductance and photosynthesis, whereas more severe deficits led to a reduction. Buckley [23] reviewed that with increased water loss or decreased water supply, stomata initially open before eventually closing, primarily due to uniform changes in water potential in epidermal and guard cells affecting epidermal turgor. According to the effects of VWC on TR via SM analysis, this two-phased TR pattern suggests that stress-induced root signals from different regimes of soil drought have distinct impacts on the shoot, potentially providing the most adaptive strategy for the plants.

## Final remarks

The increasingly popular FPP platforms not only facilitate the generation of extensive functional phenotypic data but also enable precise sampling of plant tissues for producing biologically meaningful multi-omics data [24]. To tackle the challenge of decoding vast datasets, we propose converging FPP with FM and its derived, more comprehensive SM to unravel genotype–phenotype associations. This convergence will provide a novel avenue to decipher a comprehensive picture of the genetic landscape of complex physiological traits that determine the yield and fitness of a plant under both stressed and non-stressed conditions. However, it is noteworthy that there are methodological challenges for general physiologists and "phenotypers" to implement rigorous mathematical and statistical approaches for resolving differential equations in SM. Nevertheless, we remain optimistic that collaboration among scholars from various disciplines and the rapid advancements in the field of artificial intelligence will help address these challenges.

## Supplementary Material

Web_Material_uhae256

## Data Availability

The data underlying this article are available in the GitHub repository, at https://github.com/suntingsd/System-mapping.
