# Supplementary material for: Converging functional phenotyping with systems mapping to illuminate the genotype–phenotype associations
Source: Hortic Res. 2024 Sep 9;11(12):uhae256. doi: 10.1093/hr/uhae256 (PMC11630247; doi:10.1093/hr/uhae256)
Supplement: Web_Material_uhae256 [file web_material_uhae256.zip › Table S1.docx]

| **Table S1. SNPs associated with the competition-cooperation between VWC and TR as identified by SM analysis at FDR < 0.05.** | | | | | | |
| --- | --- | --- | --- | --- | --- | --- |
| **SNP** | **Allele** | **Chr** | **POS** | **FDR** | **Gene ID** | **Annotation information** |
| 2_22160 | G/A | Vu01 | 1476294 | 0.003834 | Vigun01g013200 | VPS26A vacuolar protein sorting 26A |
| 2_01924 | C/T | Vu01 | 29205806 | 9.17E-05 | Vigun01g115400 | NB-ARC domain-containing disease resistance protein |
| 2_18925 | A/C | Vu02 | 29718001 | 0.003834 | Vigun02g150200 | Phototropic-responsive NPH3 family protein |
| 1_0248 | A/C | Vu02 | 33325722 | 0.009763 | Vigun02g198200 | ATPMEPCRA, PMEPCRA methylesterase PCR A |
| 1_0039 | G/A | Vu02 | 33344656 | 0.009763 | Vigun02g198500 | UBQ10 polyubiquitin 10 |
| 2_11823 | G/A | Vu03 | 354969 | 2.26E-05 | Vigun03g005100 | ATMPK15, MPK15 MAP kinase 15 |
| 2_05820 | G/A | Vu03 | 47358770 | 0.002505 | Vigun03g290600 | XTH7 xyloglucan endotransglucosylase/hydrolase 7 |
| 2_17846 | C/T | Vu03 | 60059279 | 2.63E-06 | Vigun03g394400 | / |
| 2_45722 | A/G | Vu04 | 11187711 | 0.006596 | / | / |
| 2_42400 | C/T | Vu05 | 23478346 | 3.00E-06 | / | / |
| 1_0029 | A/G | Vu05 | 47243288 | 0.003487 | Vigun05g284300 | CS17, PRPS17, RPS17 ribosomal protein S17 |
| 2_19025 | G/T | Vu06 | 28292796 | 0.001916 | Vigun06g160400 | F-box family protein with a domain of unknown function (DUF295) |
| 2_21882 | G/A | Vu06 | 28302945 | 0.001916 | / | / |
| 2_29599 | G/A | Vu07 | 22947436 | 0.009209 | Vigun07g124100 | Late embryogenesis abundant protein (LEA) family protein |
| 2_16551 | C/T | Vu07 | 36348834 | 0.009209 | Vigun07g242100 | ATLACS6, LACS6 long-chain acyl-CoA synthetase 6 |
| 1_0037 | A/G | Vu08 | 34929771 | 0.010455 | Vigun08g179400 | ACAM-2, CAM5 calmodulin 5 |
| 1_0733 | A/G | Vu08 | 37875535 | 2.82E-07 | Vigun08g219600 | emp24/gp25L/p24 family/GOLD family protein |
| 1_0076 | G/A | Vu09 | 30087200 | 0.006181 | Vigun09g139200 | NACA2 nascent polypeptide-associated complex subunit alpha-like |
| 1_0002 | G/A | Vu10 | 37586764 | 0.003487 | Vigun10g156100 | RPT1A regulatory particle triple-A 1A |
| 2_46358 | C/T | Vu011 | 10162946 | 0.006181 | / | / |
| 1_0082 | G/A | unmapped | / | 2.82E-07 | / | / |
| 1_0085 | G/A | unmapped | / | 0.010455 | / | / |
| 2_07104 | C/T | unmapped | / | 0.002505 | / | / |
| 2_45878 | C/T | unmapped | / | 5.14E-07 | / | / |
